# Improving Mitochondrial Function Protects Bumblebees from Neonicotinoid Pesticides

**DOI:** 10.1371/journal.pone.0166531

**Published:** 2016-11-15

**Authors:** Michael B. Powner, Thomas E. Salt, Chris Hogg, Glen Jeffery

**Affiliations:** 1 University College London, London, United Kingdom; 2 City, University of London, London, United Kingdom; 3 Neurexpert Ltd, London, United Kingdom; University of Cologne, GERMANY

## Abstract

Global pollination is threatened by declining insect pollinator populations that may be linked to neonicotinoid pesticide use. Neonicotinoids over stimulate neurons and depolarize their mitochondria, producing immobility and death. However, mitochondrial function can be improved by near infrared light absorbed by cytochrome c oxidase in mitochondrial respiration. In flies, daily exposure to 670nm light throughout life increases average lifespan and aged mobility, and reduces systemic inflammation. Here we treat bumble bees with Imidacloprid a common neonicotinoid. This undermined ATP and rapidly induced immobility and reduced visual function and survival. Bees exposed to insecticide and daily to 670nm light showed corrected ATP levels and significantly improved mobility allowing them to feed. Physiological recordings from eyes revealed that light exposure corrected deficits induced by the pesticide. Overall, death rates in bees exposed to insecticide but also given 670nm light were indistinguishable from controls. When Imidacloprid and light exposure were withdrawn, survival was maintained. Bees and insects generally cannot see deep red light so it does not disturb their behaviour. Hence, we show that deep red light exposure that improves mitochondrial function, reverses the sensory and motor deficits induced by Imidacloprid. These results may have important implications as light delivery is economic and can be placed in hives/colonies.

## Introduction

Bees play a key role in pollination critically underpinning the world’s agricultural economy. However, their number has declined significantly, posing a threat to food production. This has been linked to use of neonicotinoid insecticides that can remain effective in the soil for long periods and are a persistent threat to bees [1]. Neonicotinoids work by increasing neuronal sensitivity to acetylcholine receptor activation that over stimulates nerve cells, and subsequently depolarization of the cells mitochondria that are their primary energy source. This results in insect immobility and subsequent death [2,3]. Mitochondria produce adenosine triphosphate (ATP) via the electron transport chain, which is the molecular unit of currency for energy that underpins cellular function. Mitochondrial membrane potentials and ATP production decline with age and disease and this can signal cell death [4,5,6,7].

Compromised mitochondrial membrane potentials and reduced ATP production can be corrected. Cytochrome c oxidases in complex IV of the mitochondrial respiration chain absorbs specific long wavelengths of light [8] and in turn this improves cellular respiration [9,10]. Hence, brief exposures to 670nm light in old mice and murine models of disease increases mitochondrial membrane potentials [11] and ATP production [9,12] and this reduces disease based and age related inflammation [11,13,14]. Similar impacts are found in insects. However, here in the relatively short lived Drosophila, 670nm light exposure significantly extends average lifespan and improves aged mobility as well as reducing age related inflammation and lifting ATP production in old flies [15]. As neonicotinoid insecticides undermine mitochondrial function and reduce mobility [2,3], we ask if exposure to such long wavelength light can be used to combat their toxic effects in bees. We quantify deficits in ATP, mobility, visual function and survival in bees exposed to Imidacloprid, which is a widely used neonicotinoid pesticide and show significant correction in these deficits with brief daily 670nm light exposure.

## Materials and Methods

Bees (Bombus terrestris audax) were obtained from commercial hives (Koppert UK), although the correct nomenclature for a group of bumblebees is a colony not a hive. Bees from these were transferred to transparent 3L plastic containers and maintained on 50% sucrose solution under standard 12/12 light dark cycle with pollen supplied. Four groups were established during an intervention period that ran from Day 0 to Day 10 and a recovery period that ran from D10 to D32 where 670nm light and Imidacloprid were withdrawn. Survival numbers and mobility were recorded daily at approximately 10 am.; 1. Controls with no intervention. 2. Those given 10nM Imidacloprid in 50% sucrose. 3. Those given 10nM Imidacloprid in 50% sucrose and exposed to 670nm light twice daily for 15mins at a total of 40mw/cm2 from two light sources at either end of the container. 670nm was delivered by specific 670nm light emitting LEDs. The spectral and energy output of these were checked before and after use. 4. Those exposed to 670nm alone for the same time and intensity. All light treated bees were exposed for 15 min to 670nm light prior to Imidacloprid administration. All groups contained >100 bees. These were generally divided into groups with approximately 10 bees in each 3L container. These animals were used to assess visual function mobility and survival.

### ATP measurements

To determine the impact of 670nm light on mitochondrial function in each of the 4 groups of bees, ATP was measured in each. Whole body bee ATP was measured using a commercially available ATP determination kit (Life Technologies). Four bees were used from each of the four groups. Bees were snap frozen and homogenised in 6M guanidine HCl and snap frozen prior to ATP determination. Three technical replicates containing one bee were used for each group. Total ATP levels were normalised for total protein levels, determined with a BCA protein assay (Thermo Scientific). Data were normalised against controls.

### Mobility

Bees were categorized as mobile if free moving or initiated movement in response to mechanical stimulation of the body. They were categorized as alive if any limb movement was present in response to stimulation. Data was gathered over 31 days and for both mobility and survival it was normalized to controls on Day 0.

### Visual function

Surviving bees from each group were immobilised on a glass slide with tape and a glass micropipette (tip diameter 5–10μm filled with physiological saline) placed just below the corneal surface in one eye on day 5. A steel indifferent (earth) electrode was inserted into the thorax. Visual stimulation was 500ms white light flashes at 0.3W/m2. Amplitudes of the response were recorded via an Axoprobe 1A amplifier, digitized (5kHz) by a CED1401 interface and stored on a PC. Peak to peak single responses of the first flash presented in a train were recorded for each bee, and mean values were computed for each bee and treatment group. Statistical analysis was by ANOVA with post-hoc t-tests.

In addition to these experiments, two Koppert commercial hives were exposed to insecticide via the water supply at the same concentration as above and one of these was exposed to 40mw/cm^2^ of 670nm as above. These had a Perspex top and as they were kept in low ambient illumination the bees did not attempt to cover their colony but left its core exposed. Because of the high level of activity of the animals, the majority of bees in the 670nm exposed colony spent a proportion of the time under 670nm light. The activities of both colonies were filmed.

## Results

### ATP measurements

**Fig 1:**
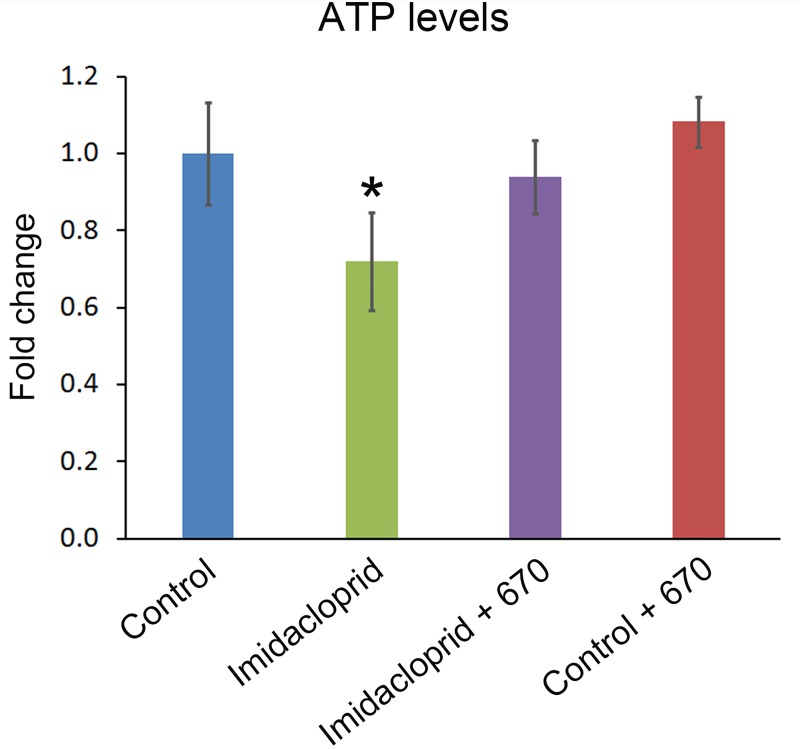
ATP measurments. ATP levels were measured in 4 animals in each group and the data normalised to controls. Exposure to Imidacloprid resulted in a statistically significant reduction in ATP of around 30% in comparison to each of the other groups. However, in bees exposed to Imidacloprid and 670nm light, ATP levels were significantly improved and satatistically similar to controls. In bees exposed only to 670nm light there was an increase in ATP over levels found in controls of about 10%, but this was not statistically significant. These data reveal that Imidacloprid undermines mitochondrial function, but that this is corrected by exposure to 670nm light. Error bars are SEM. Symbol * p<0.05.

Fig 1 shows that ATP declined by a significant 25% in bees exposed to Imidacloprid compared to controls (p<0.05). However in those bees that were also exposed to 670nm, ATP levels improved significantly over the Imidaclopride alone treated group, returning close to control levels (p<0.05). In control bees only exposed to 670nm, ATP levels increased over controls by approximately 10%, but this was not statistically significant. However, there remained a significant difference between this group and those treated with against Imidacloprid alone (p<0.05). These data demonstrate that Imidacloprid undermines mitochondrial function but that this is corrected by 670nm light.

### Bee survival

**Fig 2:**
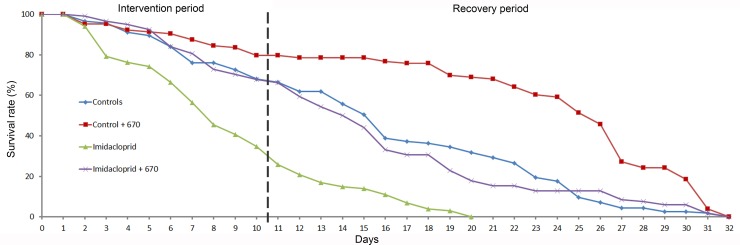
Survival rates. Bees were divided into 4 groups with >100 animals in each. Survivors were counted daily over 32 days. Data were normalized to controls on day 0. This period was divided into an intervention time (0–10 days) and a recovery time (11–32 days). The four groups were untreated controls, controls given 670nm light, those given Imidacloprid, and finally those given Imidacloprid and 670nm light. Both Imidacloprid and 670nm light were withdrawn in the recovery period. Imidacloprid treated bees showed a rapid decline even in the recovery period with a 70% loss rate against only 25% in controls in days 0–10. All were dead by day 20. There were no differences between controls and Imidacloprid treated plus 670nm in either the intervention or recovery period. Bees exposed to 670nm alone had a loss rate of approximately 20% during the period intervention period. However their rate of decline was significantly lower than the other 3 groups in both periods. Hence, exposure to 670nm in Imidacloprid treated bees corrects death rates result from insecticide exposure. Further, its application can have a protective effect for periods after its use is withdrawn.

Fig 2 shows bee survival rates divided into 2 periods. The first period was the intervention period (0–10 days), during which Imidacloprid was administered to two groups of bees, one of which was also exposed to 670nm light. A separate group was given 670nm light alone and a final group was a control that received neither light nor insecticide. The second period was the recovery period running from 10–32 days. Here the same groups were continued but insecticide and light treatment were withdrawn. There were no brood cells in the containers.

During the intervention period control bees had a steady death rate over the 10 days with approximately 30% of the animals dead by day 10. The imidacloprid treated group also showed a steady loss rate but this was significantly increased over controls (P<0.001). Increased death rates in Imidacloprid treated bees over control animals started at day 3 when numbers were reduced by 20%. By day 7 death rates here were >50% and reached 70% by day 10. Exposure to 670nm light had a positive impact on death rates in Imidacloprid treated bees as these were significantly reduced compared to Imidacloprid alone (P<0.001), but not different from controls (P<0.49). Treatment with 670nm on control bees also resulted in a significant improvement in survival over controls (P<0.01), similar to data from fruit flies exposed to this light [15], with around a 15% loss rate over the 10 days. Hence, 670nm exposure protected against Imidacloprid induced death.

During the recovery period when Imidacloprid and 670nm light were withdrawn, death rates continued in bees that had been exposed to insecticide, although the gradient of bee loss was reduced. However, all of these bees were dead by day 20. Death appeared to be due to reduced mobility resulting in an inability to feed and perhaps also to the lack of cleansing flights in the restricted environment of the insecticide exposed animals that may have differentially impacted upon them. Rates of death in controls and Imidacloprid + 670nm light continued at a rate similar in the recovery period to that in the intervention period. There was no statistically significant difference between these two groups of animals who were all dead by day 32, but there was a significant difference between these two groups and the Imidacloprid treated group (P<0.001). Control bees exposed to 670nm alone maintained a significantly reduced rate of death over the entire recovery period in relation to the 3 other groups (P<0.001), demonstrating that 670nm light treatment had a sustained impact long after it was withdrawn. However, as with flies treated with 670nm light on a similar regime, it did not increase absolute life expectancy [15].

### Bee mobility

**Fig 3:**
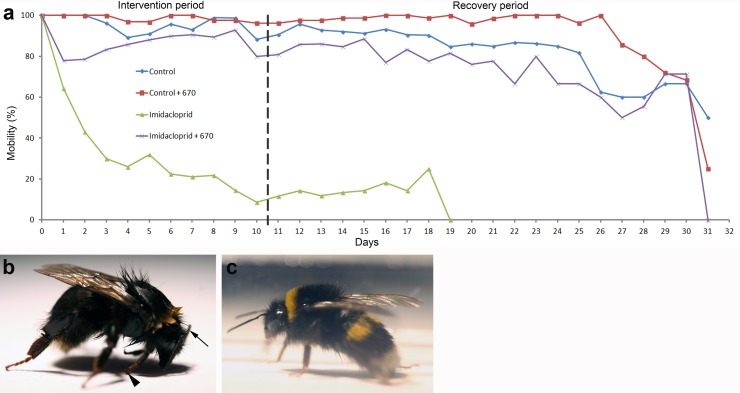
Bee mobility. The percentage of live bees that are mobile and able to walk independently or in response to mechanical stimulation is shown in 2A. Measurements were made daily in each of the four groups with data normalized to controls on day 0. Each group contained a minimum of 100 bees on day 0. Controls suffered a 10% mobility decline over the 10 day intervention period and then a gradual decline through the recovery period. There was a rapid decline in Imidacloprid treated animals of >50% by day 2 and then a gradual decline through the rest of the intervention and recovery periods. Imidacloprid treated bees exposed to 670nm suffered a 20% initial decline but by day 3–4 had recovered to a range similar to controls. Overall these animals had a slightly less mobility than controls from day 5/6 through to day 30 of around 10%. Bees exposed to 670nm light alone showed only a marginal decline of approximately 5% over 30 days. Fig 2B show a representative bee from groups exposed to Imidacloprid on day 4. It has a hunched posture with head down. Also the front legs are turned back (Arrow head) and the antenna are withdrawn towards the head. These animals did not groom and hence had a shabby appearance. Fig 2C shows an Imidacloprid treated bees exposed to 670nm. The majority of these bees were indistinguishable from controls. Because they were highly active they were difficult to photograph.

The percentage of bees retaining mobility is shown in Fig 3A. Control bees showed minimal reductions in their mobility over approximately 25 days. The maximum number of bees that were immobile in this group was 10%, but this varied on a day to day basis. Bees fed Imidacloprid suffered a marked progressive loss of mobility that was significantly greater than seen in controls (P<0.001). By day 2 the number of mobile bees in the Imidacloprid treated group declined by >50% and continued to decline until day 10 when the number showing mobility in those that survived was reduced by 95%. From day 10 onward, mobility levels stabilised until all bees in this group were dead on day 19. Many of the animals that were still mobile after day 2 showed rear leg dragging, sluggish locomotion and had an arched posture compared to controls (Fig 3B). The total distance that they traveled was very restricted and often lacked direction. However, a small minority (5–8%) were relatively unaffected. A key factor in the reduced mobility was that these animals did not feed or groom. Bees exposed to Imidacloprid and 670nm light remained largely mobile and this mobility was significantly greater than that found with Imidaclorpid alone (P<0.001). The maximum number that were immobile in this group reached 20% on days 1–2, but with no leg dragging or arched body posture. From day 3 onward mobility patterns in this group improved and roughly mirrored that in controls with a general reduction of approximately 10 until approximately day 25. However, because of the initial decline in mobility in Imidacloprid and 670nm treated animals in days 1–2, they remained significantly different from controls (P<0.05), reflecting the fact that within this experimental paradigm complete mobility had not been corrected in early insecticide exposure. Likewise, there remained a significant difference between Imidacloprid and 670nm compared to 670nm light exposure alone (P<0.001). The improved mobility in these animals allowed them to feed and groom. Our criteria for mobility were that they were free moving or responded to mechanical stimulation. This described bees that were functional animals. Differences were still apparent in other metrics that were not probed including general probability in independent initiation of movement, which remain reduced after light treatment, however, the variability in this feature between individuals was very large.

Fig 3B shows a representative example of a live bee that was mobile after imidacloprid exposure. The fore legs are turned back (large arrow head) and the antenna are withdrawn along the head (smaller arrow). The body posture is arched with the head down and the bee has failed to groom resulting the fur sticking together and loss of its yellow colouration. These animals did not fly within the boxes. Fig 2C shows an Imidacloprid treated bee exposed to 670nm light. The majority of bees in this group were indistinguishable from controls with normal posture and grooming. They also had the ability to fly within their boxes.

### Bee visual sensitivity

Having established that 670nm light normalises survival and improves compromised mobility we ask if Imidacloprid undermines visual function and if this can also be ameliorated by 670nm. Vision is a primary sensory modality for bees, critical in both navigation and flower selection. Their visual range spans 300-650nm with significant ability to discriminate wavelengths [16,17].

**Fig 4:**
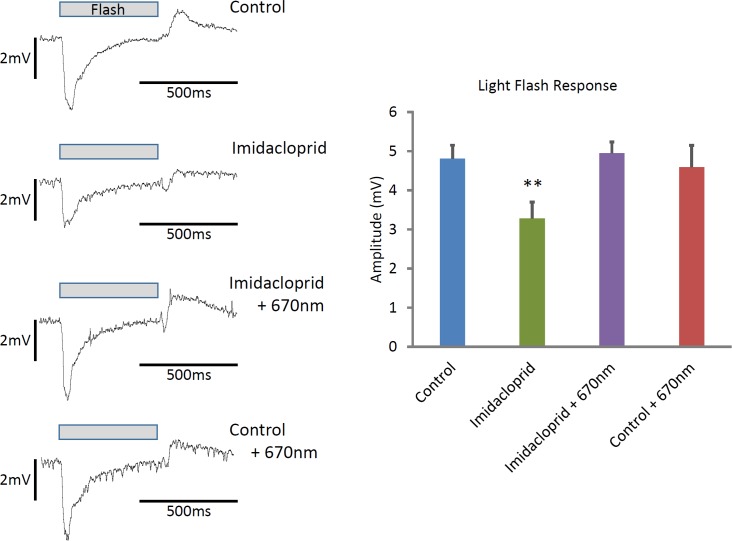
Visual function. Electrophysiological recordings from the eye on day 5. Traces of example individual waveforms from each group are shown on the left side of the figure. There were no significant differences in responses from bees between controls, Imidacloprid plus 670nm, and 670nm alone with all having an approximate 5mV peak to peak response. However, Imidacloprid treatment on its own resulted in a significant reduction in this response to approximately 3mV compared to the other groups. Imidacloprid treated bees have a marked reduction in both response peaks. Hence, Imidacloprid treated bees have significant reductions in retinal function that were corrected by 670nm light exposure. On the right side of the figure these data are represented as histograms giving the group means (± s.e.m.). (Control N = 9, Imidacloprid N = 6, Imidacloprid +670nm N = 6, Control +670nm N = 11; ** P<0.01 compared to other groups).

The bee retinal response has two key components, an initial negative deflection at stimulus onset followed by a positive wave at stimulus offset similar to previously described responses [17]. Here we measure the amplitudes of the peak to peak responses. Fig 4 shows magnitude of the ERG responses in the waveforms from the four groups. In controls this was approximately 5mV, with a 3.75mV negative wave followed by a positive wave of around 1.25mV. In Imidacloprid treated bees both waves were significantly reduced with a combined magnitude of response just over 3mV, confirming that visual function was compromised in these bees. Notable in this was the approximately 30% reduction in the size of the negative wave. In Imidacloprid + 670nm treated bees and those treated with 670nm alone, the peak to peak response was the same as in controls. The only statistically significant differences were between the Imidacloprid treated group and the other 3 groups (P<0.01). The right had side of the figure shows graphically the magnitude of the responses in the groups. It is likely that the improved physiological responses from the retina are due to increased availability of ATP for Na and K pump responsible for maintaining photoreceptor membrane potentials. Hence, 670nm light is able to correct both sensory and motor induced damage in Imidacloprid exposed bees.

Filming of bee activity in the commercial hives is shown in supplementary material (S1 Video). Bees in the group exposed to insecticide were similar to those described above. They did not fly and were silent even in response to tapping of the side of the box that contained the bees. They tended to group in a disordered manner. Bees in the 670nm treated group were indistinguishable to controls. They flew and had a normal appearance. They were very active and there was a continued background buzzing and a strong auditory response was obtained if the box was tapped.

## Discussion

Our results reveal that 670nm light treatment significantly reduced bee death rates and improved ATP levels, mobility and visual function in animals exposed to Imidacloprid. 670nm light is known to improve mitochondrial function that has declined with age and disease [9,10,11,12,13,14,15]. Although there was an initial small drop in mobility in Imidacloprid exposed bees given 670nm light, these animals remained largely functional and able to groom and feed. We have maintained bees for >30 days in experimental conditions with insecticide and light, which is considerably longer than most laboratory investigations examining the impact of neonicotinoids. However, we have divided this into two periods removing the insecticide and the light treatment in the second window. These data reveal that the impact of 670nm on these animals continues after the light is withdrawn. This also demonstrates that the effects were not due to warming, which was minimal. While the results of 670nm treatment were positive, we have found in additional pilot studies that they are less so when used on bees from colonies that had already started to show significant signs of decline. But they have an increased positive impact when treatment includes a prophylactic element, exposing bees to 670nm prior to the insecticide. Further, we have also shown that improved mobility and survival is found when 670nm lighting is introduced directly into experimental colonies exposed to this insecticide (see S1 Video). Both in bees removed from colonies and those treated within them, Imidacloprid suppressed buzzing, while treatment with 670nm light returned this behaviour to normal.

The true impact of this deep red light is probably much greater than revealed here. We have made no attempt to vary light energy, duration, time of day of the exposure or the wavelength to optimize effect. Other wavelengths are known to be effective via COX absorption and include approximately 810nm and around 1000nm [9,10,14]. Likewise, our dosages of insecticide maybe higher than experienced in the field as bees in the wild are likely to sample both sources contain insecticide and those that do not. However, it is also possible that neonicotinoids translocate to wild flowers and as such extend the period and sources of exposure. Taken together this implies that there is considerable room to improve efficacy and that the real challenge may be smaller than presented here.

Exposure to 670nm has proved highly successful where mitochondrial function is undermined by ageing, disease or induced pathology. The retina has been a key target of this treatment in mammals because of its high energy demand and the fact that with ageing there is significant reduced function and high cell loss [11,12,13,14]. However, it has also proved highly effective in the brain where energy demands are also high [18]. Recently, it has been used successfully in diverse situations including treatment of experimental Parkinson’s disease. In fly models of Parkinson’s, 808nm has been shown to improve complex IV dependent respiration, ATP production and reduce flight defects [19]. In primates where drugs that initiate the disease target mitochondria in the brain stem, 670nm light delivered via fiber optics specifically to this region when disease is initiated, results in significantly fewer clinical signs [20]. These studies that span widely separated species are important and relevant because immobility is a key feature in Imidacloprid exposed bees, and near infrared light impacts positively on both mammals and insects in this respect. In spite of this these results need to be confirmed in a wider range of insects including honeybees.

Light sources delivering 670nm have commonly delivered between 20-40mW/cm^2^ for between 1-30mins [14]. These longer wavelengths deeply penetrate the mouse brain and likely penetrate the whole body of bees used in the current study. It is likely that such penetration impacts widely on the bee CNS, and as such it is probable that this may impact on higher order functions including navigation. However, as with some previous mouse studies [12,13], we made no attempt to restrain animals, rather they were free to move within their housing and in relation to the light source. Hence, effective dosing energy and time of exposure probably have a relatively wide spectrum, although there is evidence that excess exposure has reduced efficacy [18]. These features are important as we are prototyping economic 670nm light source devices for colonies and we have confirmed that the overall pattern of protection we reveal here can be obtained when insecticide exposed colonies are illuminated with 670nm as in the video data shown in the supplementary material. While it is clear that such light sources will positively impact on bees exposed to neonicotinoid insecticides, they have the added advantage of being beyond the bee’s visual range and so should not disturb normal behaviour [16,17].

## Supporting Information

S1 Video (MP4)
